# Toxicity Profile of the Aqueous Ethanol Root Extract of *Corrigiola telephiifolia* Pourr. (Caryophyllaceae) in Rodents

**DOI:** 10.1155/2011/317090

**Published:** 2010-09-07

**Authors:** Hind Lakmichi, Fatima Zahra Bakhtaoui, Chemseddoha A. Gadhi, Aicha Ezoubeiri, Younes El Jahiri, Abdellah El Mansouri, Ibtissam Zrara, Kenza Loutfi

**Affiliations:** ^1^Unit of Phytochemistry and Pharmacology of Aromatic and Medicinal Plants, Laboratory of Biotechnology, Protection and Valorization of Plant Resources, Faculty of Sciences Semlalia, Cadi Ayyad University, Marrakech 40000, Morocco; ^2^Laboratory of Plant Physiology, Faculty of Sciences Semlalia, Cadi Ayyad University, Marrakech 40000, Morocco; ^3^Laboratory of Medical Analysis, Ibn Tofail Hospital, CHU Mohamed VI, Marrakech 40000, Morocco; ^4^Laboratory of Biochemistry, Avicenne Military Hospital, Marrakech 40000, Morocco; ^5^Laboratory of Anatomo-Pathology, Avicenne Military Hospital, Marrakech 40000, Morocco

## Abstract

*Corrigiola telephiifolia* Pourr. (Caryophyllaceae) is a Moroccan medicinal plant. Despite its popular usage, no study has been published concerning its toxicological profile. The acute toxicity of *C. telephiifolia* root extract was evaluated by giving it orally to mice at single doses of 5000, 10000, and 14000 mg/kg bodyweight. The extract was also administered at doses of 5, 70, and 2000 mg/kg bodyweight per day to rats for a forty-day toxicity study. No mortality or signs of toxicity were observed in the acute study. In the forty-day study in rats, the extract at 5 mg/kg/day showed no toxicological effects in either sex. At 70 mg/kg/day, the treated group differed from the control only by a significant decrease in serum concentrations of sodium and chloride ions (*P* < .05). At the dose of 2000 mg/kg/day, the extract significantly increased the serum concentrations of creatinine, alkaline phosphatase, gamma-glutamyltransferase and phosphorus (*P* < .05) all suggestive of functional nephrotoxicity and hepatotoxicity. The relative bodyweight of both sexes decreased at the dose of 2000 mg/kg/day, with a fast recovery for males. Histological examination did not reveal any treatment-related effects. In conclusion, Corrigiola extract appears safe at the doses used ethno-medicinally. Much higher doses pose toxicological risks.

## 1. Introduction

Plants which are commonly used in traditional medicine are frequently promoted as natural and, therefore, harmless. This assessment is based on their usage in the treatment of diseases over centuries [1, 2]. However, some medicinal plants must be used with caution because they can cause adverse reactions, especially if they are taken in excessive doses, or if they interact with conventional drugs [3–13]. Consequently, in response to public health concerns, research that focuses on deficiencies in the knowledge about medicinal plants and their potential toxicities is highly encouraged by many official medical and scientific organizations [14–17] and by complementary and alternative medicine (CAM) researchers and practitioners [18, 19].

In Morocco, the use of medicinal plants is an important and essential part of the culture and of the traditional healthcare system. However, the production, prescription, and use of these plants are not currently regulated. As a result, there is always the danger of inappropriate use, incorrect identification, and interactions with concurrently administered drugs.

*Corrigiola telephiifolia* Pourr. (Caryophyllaceae) is a Moroccan medicinal plant called “*Sarghina.*” It is a herb, widely branched from the base, with slender prostrate branches and tiny compact inflorescences. The root, which is used for medicinal and cosmetic purposes, is a perennial tap root. This species is found in Southern Europe and North Africa. In Morocco, it grows in cultivated beds on rocky and sandy soils. It is widely spread in the Atlas and Rif mountains [20]. Morocco exports annually a quantity of about 370 tons [21]. When burned, the root of this plant releases an aromatic fume. The root is also used to treat flu, dermatological diseases, inflammation, ulcer, cough, and jaundice; it is also used as an anasthenic and a diuretic [20]. *Corrigiola *root is part of a traditional remedy given to parturient women. The powdered root is traditionally consumed plain, mixed with honey, or simply sprinkled on food ([22], personal investigation). 

Despite the exposure of the Moroccan population to this plant and particularly its use by many women in delicate health conditions (e.g., postpartum period), there is no information in the scientific literature on its toxicity profile, its chemical composition, or its pharmacological properties, except for one orphan report on its anti-inflammatory activity [23].

The present investigation was therefore undertaken to evaluate the potential toxic risks incurred following the ingestion of hydroalcohol extract of *Corrigiola telephiifolia* in rodents.

## 2. Methods

### 2.1. Preparation of *Corrigiola telephiifolia* Extract

*Corrigiola telephiifolia* Pourr. (Caryophyllaceae) was collected during its blossoming stage in June 2003 from Ben Slimane in western Morocco. The whole plant was identified by Professor A. Ouyahya, a taxonomist from the Scientific National Institute (Rabat); A voucher specimen (N° RAB65892) was deposited in the Botany Department.

The roots were separated from the aerial parts of the plant, washed, cut into small pieces, and dried under shade. They were thereafter ground into a powder. A quantity of the powder (400 g) was exhaustively extracted with ethanol-water mixture (75 : 25) in a Soxhlet extractor. The aqueous ethanol extract was concentrated to dryness under vacuum. The residue (yield = 33% w/w) was stored in a refrigerator at 4°C until the time of drug administration.

### 2.2. Phytochemical Screening

Preliminary phytochemical screening of the extract involved qualitative determinations of the following substances: alkaloids, saponins, terpenes, tannins, quinones, and flavonoids. Determinations were carried out in accordance with procedures described by Harborne [24]. 

### 2.3. Experimental Animals

Adult Swiss albino mice and Wistar rats of either sex were used for the acute and forty-day toxicity studies, respectively. The animals were supplied by the Animal Care Facility of the Faculty of Sciences Semlalia, Cadi Ayyad University, Marrakech, Morocco. They were housed in groups of four animals in stainless steel cages (males separated from females) and kept under standard environmental conditions (25 ± 2°C; 12/12 h light/dark cycle). They were fed *ad libitum* with Cicalim pellets (Cicalim s.a., Casablanca, Morocco) and barley. All animals had free access to tap water. They were acclimatized for 5 days before the beginning of the study. All studies were conducted after obtaining prior approval from the institutional ethical committee in accordance with the National Institute of Health “Guide for the Care and Use of Laboratory Animals” (NIH publication no. 85-23, 1985).

### 2.4. Acute Toxicity

Before the experiment, the mice did not have access to food for four hours as recommended by the Food and Drug Administration [25]. *C. telephiifolia* extract was then dissolved in distilled water and given orally to four groups of mice (*n* = 16/dose, eight males and eight females) at single doses of 0, 5000, 10000, and 14000 mg/kg bodyweight. These doses took into account the solubility of the extract in distilled water. Animals were observed during the first 12 hours for signs and symptoms such as modifications in autonomic activity, posture, piloerection, and respiratory pattern. Other signs and symptoms observed include occurrence of hemorrhage, diarrhea, convulsions, tremors, sedation, and death. The mice were weighed daily and observed for fourteen days following treatment. At the end of the fourteen-day period, the animals were sacrificed under urethane anesthesia (1 g/kg, i.p.). The heart, liver, kidneys, pancreas, lungs, stomach, and spleen were immediately removed, weighed, and placed in Bouin's solution. After dehydrating and embedding, sections of 4-5 microns thick were cut using a rotary microtone. The sections were stained with haematoxylin and eosin and examined microscopically.

### 2.5. Forty-Day Toxicity

Sixty-four (64) rats were randomly assigned into four groups (*n* = 16), which include eight males and eight females in each group. Prior to the experiment, the animals were kept away from food overnight [26]. The first group served as control (only water given), while the remaining three groups each received daily doses of 5, 70, and 2000 mg/kg body weight of *C. telephiifolia* extract p.o. for forty days. After the last dose, rats were not fed overnight. They were anesthetized (on day 41) by intra-peritoneal injection of a dose of urethane (1 g/kg). Blood was collected in two types of tubes: one with EDTA and the other without additives. The anticoagulated blood (tube with EDTA) was analyzed immediately for hematological parameters. The second tube was centrifuged at 3000 rpm at 4°C for 10 min to obtain the serum for biochemical analysis. The animals were then sacrificed by exsanguination under urethane anesthesia and the organs were removed for histopathological analysis. 

### 2.6. Bodyweight, Mortality, and Clinical Signs

During the forty-day dosing period, all animals were weighed and observed daily for convulsions, excitement, posture, piloerection, breathing difficulty, sedation, anorexia, diarrhea, hemorrhage, and death. Observations were made immediately before dosing, and up to four hours after dosing.

### 2.7. Biochemical Analysis

Biochemical analysis of serum samples was performed using an automatic chemistry analyzer (Vitros System Chemistry 250, Ortho Clinical Diagnostics, Johnson and Johnson Company, USA). The biochemical parameters measured were sodium, chloride ion, potassium, magnesium, calcium, iron, bicarbonate ion, phosphorus, total protein, glucose, urea, uric acid, and creatinine. Others include cholesterol, triglycerides, bilirubin, aspartate-aminotransferase (AST), alanine-aminotransferase (ALT), gamma-glutamyltransferase (GGT), and alkaline phosphatase (ALP).

### 2.8. Hematological Analysis

Hematological analysis was performed using an automatic hematological analyzer (ABX MICROS 60-OT). The hematological parameters measured were white blood cell count (WBC), red blood cell count (RBC), platelets (PLT), red cell distribution width (RDW), hemoglobin (HGB), hematocrit (Hct), mean corpuscular volume (MCV), mean corpuscular hemoglobin (MCH), mean corpuscular hemoglobin concentration (MCHC), red cell distribution width (RDW), and mean platelet volume (MPV).

### 2.9. Autopsy and Microscopic Examination

Macroscopic examination of vital organs was carried out soon after sacrifice. Different organs, namely, the heart, liver, kidneys, stomach, lungs, and spleen were surgically removed, placed on absorbent papers for a few minutes, and then weighed (absolute organ weight in grams). The relative organ weight (ROW) of each animal was then calculated as follows:

(1) $$\text{ROW}=\left[\frac{\text{Absolute organ weight}\left(g\right)}{\text{Bodyweight}\;\text{of}\;\text{rat}\;\text{on}\;\text{sacrifice}\;\text{day}\;\left(\text{g}\right)}\right]\times 100.$$

Tissue biopsies from excised organs were fixed in Bouin's solution. Following dehydration and embedding, sections were cut at 4-5 microns with the rotary microtone, stained with hematoxylin and eosin, and examined microscopically to assess any potential toxic effects [27]. 

### 2.10. Statistics

Results are expressed as mean ± standard error of mean (S.E.M). Significant differences between control and experimental groups were assessed by Student's t-test. Differences were accepted as significant at *P* < .05.

## 3. Results

### 3.1. Acute Toxicity in Mice

The oral administration of a single dose (5000, 10000, and 14000 mg/kg bodyweight) of *C. telephiifolia* extract to mice did not cause death within the fourteen days of the study (Table 1). No signs of toxicity or significant bodyweight changes were recorded. The histopathological examination (not presented) of selected organs showed normal architecture similar to the control group. These observations reveal that the oral medium lethal dose value (LD_50_) of the hydroethanol root extract of *C. telephiifolia *is greater than 14000 mg/kg bodyweight in mice.

### 3.2. Forty-Day Toxicity in Rats

#### 3.2.1. General Observations

Throughout the 40-day feeding study, there was no mortality recorded in either the control or treated groups at the administered doses (Table 2). Rats treated with a dose of 5 mg/kg/day behaved normally, apart from slight abdominal contractions observed during the first week after the extract administration. The clinical signs observed in rats treated with an extract dose of 70 mg/kg/day were mainly abdominal contractions (observed after gavage), reduced activity, hunched posture, loss of appetite, and mild diarrhea. These clinical signs lasted one or two weeks depending on the sex (females seemed more susceptible than males) (Table 2).

At the dose of 2000 mg/kg/day, animals, especially females, seemed more affected. Pronounced clinical signs such as abdominal contractions, inactivity, prostration, intense diarrhea, and anorexia were observed in all animals. In addition, respiratory complications were noted in two rats. These observations were made during the initial three to four week period. During the exposure period, setting aside sex and dose (70, and 2000 mg/kg bodyweight), signs of polyuria were noted among the treated animals and their feces had taken on the yellow color of the plant extract (Table 2).

#### 3.2.2. Bodyweight

The mean bodyweights of the animals in the study are presented in Table 3. Weight gain was common and significant in both sexes of the control rats and those given 5 or 70 mg/kg/day. Animals treated with 2000 mg/kg/day showed a significant weight loss compared with the control groups. For males, this reduction in weight was statistically significant during the first week. These male rats subsequently recovered their weight, but female rats did not recover their weight throughout the treatment period. 

#### 3.2.3. Hematological and Biochemical Parameters

The hematological parameters were not significantly different between the treated rats and the control group (Table 4). The values of the biochemical parameters of female and male rats are shown in Tables 5 and 6, respectively. 

The doses of 5 mg/kg/day and 70 mg/kg/day did not cause any significant changes in the measured parameters except for sodium and chloride ions, which were significantly decreased in the rats that received 70 mg/kg/day.

At the dose of 2000 mg/kg bodyweight, in addition to a decrease in sodium and chloride ions observed in both sexes, the treated females showed significant increase in creatinine, phosphorus, ALP, and GGT. Total proteins decreased significantly when compared with those of the control group (Table 5).

#### 3.2.4. Autopsy and Microscopic Examination

The macroscopic observation of the organs did not present any significant morphological or hemorrhagic changes due to the administration of the extract. There were also no statistically significant differences in the relative organ weights (Table 7). Histopathological examination of the different organs demonstrated that there were no pathological features observed in either the control or the treated groups.

### 3.3. Phytochemical Screening of the Extract

The phytochemical screening of *C. telephiifolia* extract revealed the presence of saponins and terpenes. Alkaloids, tannins, quinones, and flavonoids were not detected.

## 4. Discussion

*C. telephiifolia* is traditionally used to treat different illnesses without any reported toxic signs. It is considered safe by Moroccan folk ([22], personal investigation). The undertaken acute toxicity study has apparently confirmed this reputation. The oral LD_50_ value in mice which is higher than 14000 mg/kg falls into class 5 of the globally harmonized classified system for chemical substances and mixtures [28].

Since the acute dose study could not provide a guideline for selecting doses for the chronic low-dose investigation, and in the absence of other toxicological data that could have helped to determine the duration of treatment, this study was designed using the WHO's recommendations for herbal medicine testing [17]. A forty-day oral toxicity study was consequently performed. This duration of the treatment corresponds in Moroccan culture to the postpartum period in which the parturient, called “nafssa,” benefits from intensive family care and other types of health care, including the use of medicinal plants. 

The doses of 5 and 70 mg/kg/day of the hydroalcohol extract p.o were also established from the results of the ethnological survey conducted within a sample of Moroccan users and herbal practitioners. These doses correspond respectively, to “a pinch” and “a spoon” of the plant powder estimated to be consumed as a remedy for an adult woman weighing 60 kg. The doses also take into account the yield (33% w/w) of *C. telephiifolia* obtained with 75% ethanol. The third dose of 2000 mg/kg/day of the hydroalcohol extract corresponds to the highest dose limit recommended by the OECD [29] to assess subchronic toxicity when there are no toxicological data available. 

Nonspecific clinical signs such as abdominal contractions, reduced activity and hunched posture were observed at a dose of 70 mg/kg bodyweight. These are probably behavioral responses to the malaise induced by the extract [29]. Mild diarrhea was noticed only during the first week of the exposure period, perhaps due to subsequent adaptation. Intestinal smooth muscle contractions may account for the observed diarrhea and the abdominal contractions. Although the smooth muscle contractions have not been characterized, they may lend credence to the use of the plant in postpartum hemorrhage.

Changes in serum sodium and chloride concentrations tend, according to the OECD guideline [29], to occur in parallel when they are associated with relative water content, and both electrolytes become depleted by fluid loss. Thus, the decrease in these two parameters might be related to the diarrhea observed during the test.

Male rats given 2000 mg/kg/day of the extract showed similar hematological and biochemical responses to those treated with 70 mg/kg/day of the extract. Histopathology was also similar. In contrast, the female groups were more sensitive and presented, in addition to decrease in Na and Cl levels, signs of nephrotoxicity, mild hepatic injury, and nutrient deficiency. These symptoms were revealed by characteristic biochemical markers such as the increase in the serum creatinine level and the phosphorus concentration for kidney injury, the increase in ALP and GGT levels for liver damage, and the decrease in serum proteins for nutrient deficiency [29–32].

Indeed, the serum protein concentration may decrease following a prolonged deficit in food consumption, a reduced protein synthesis or as a consequence of an elevated protein loss through renal dysfunction or hemorrhage [29]. In this study, the protein loss may be justified by all of these parameters, except hemorrhage. These parameters are supported by the observed clinical signs such as frequent diarrhea and bodyweight loss.

Alkaline phosphatase (ALP) is present in all tissues, but is particularly concentrated in liver, bile duct, kidney, bone, and the placenta. The increase in ALP could have been due to liver injury [27] rather than to other pathological reasons (bone disease, malignancies, etc.) because of the corresponding increased activity of GGT [29, 30, 32]. In fact, serum GGT activity is an effective indicator of hepatobiliary toxicity in rats [27, 32], and is more specific than ALP. ALP can be elevated as a result of toxic effects on bone formation while serum GGT activity is unaffected [29]. ALT is a highly sensitive biomarker of hepatotoxicity [32–39]. Although not statistically significant, the increase in serum ALT level may support the suggested hepatotoxicity.

Creatinine is formed by nonenzymatic breakdown of creatine, and changes in its serum concentration are the result of alterations in renal blood flow, renal function, or urine outflow [29, 48]. The obtained elevated blood creatinine level, which is a reliable indicator of impaired glomerular filtration in addition to the rise in serum phosphate concentration, is a sign of significant impairment of renal function [30]. The fact that a significant change in serum creatinine was observed means that a kidney injury had already developed following the oral administration of the high dose of *C. telephiifolia* [49]. 

All of these findings (nephrotoxicity, hepatic injury, and nutrient deficiency), observed at the dose of 2000 mg/kg/day in female rats, were not corroborated by histopathological damages, leading to the conclusion that the toxicological impact of *C. telephiifolia*, at this stage, is most likely functional in nature, rather than structural [50]. This assumption does not exclude the fact that in case of chronic or repeated intermittent use of the plant, more apparent deleterious signs may occur.

The reasons for the differences in the toxicity pattern of *C. telephiifolia* between male and female rats are not immediately known. They may involve factors which account for sex-related pharmacokinetic differences, that is, organ size, a higher percentage of body fat, and a lower glomerular filtration rate in females than males [51]. Of other factors, sex differences of drug-metabolizing enzymes (e.g., cytochrome P450) and/or transporter proteins (e.g., P-glycoprotein) have been considered important factors [51–57]. 

**Figure 1:**
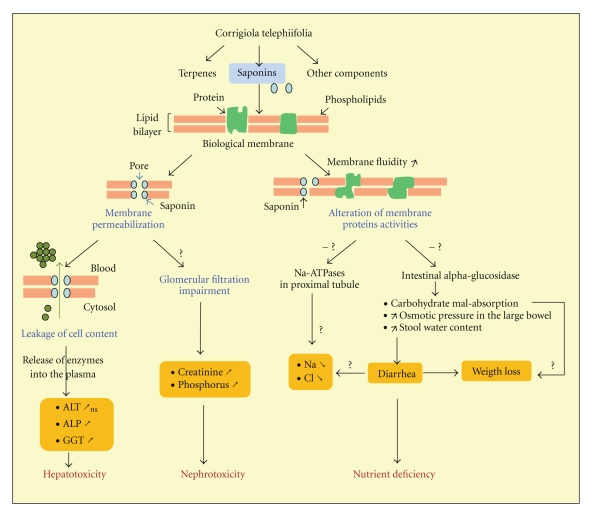
Hypothetical diagram of *Corrigiola telephiifolia* toxicity mechanism through its saponins component. The major symptoms are nephrotoxicity, hepatic injury, and nutrient deficiency. -: inhibition; ↗: increase; ↘: decrease; ?: theoretical pattern on the basis of reported activities of some saponins [40–47]; ns: no statistically significant.

The phytochemical screening of the root extract showed that the plant is rich in terpenes and saponins. Saponins are known for their wide range of biological activities [58–61]. These activities include disruption of biological membranes [61] and generation of free radicals [62] that cause lipid peroxidation [63]. Saponins make the lipid bilayer permeable to macromolecules [64] by inducing pore-like structures [61]. The subsequent increase of membrane fluidity was supposed to be one of the key steps in saponin-induced toxicity [61]. The alteration of the lipid environment around membrane proteins leads to the alteration of their activities as transporters, ion channels, and receptors [40–47, 60]. Consequently, changes in some biochemical parameters levels in both sides of the membrane (electrolytes, enzyme substrates, enzymes.) may emerge as signs of saponin-induced biological effects. This may give hypothetical explanations concerning data observed in this study (Figure 1), bearing in mind that saponins are not the only components of *Corrigiola *extract.

In conclusion, *C. telephiifolia* root extract appears safe at the doses used ethnomedicinally. Much higher doses pose toxicological risks. Therefore, it is prudent to undertake additional research in order to characterize other toxicological effects which might arise following long-term use of the extract.

## Figures and Tables

**Table 1:** Effect of single oral doses of *Corrigiola telephiifolia *extract in mice.

Dose of CT extract^a^ (mg/kg)	Sex	D/T^b^	Symptoms
0	M	0/8	None
	F	0/8	None
5000	M	0/8	None
	F	0/8	None
10000	M	0/8	None
	F	0/8	None
14000	M	0/8	None
	F	0/8	None

^a^ *C* *orrigiola telephiifolia *extract was administered as a single oral dose to groups of mice. All treated mice were carefully examined for any signs of toxicity during a period of 14 days.

^b^D/T: dead /treated mice.

Median lethal dose value (DL_50_) > 14000 mg/kg bodyweight.

**Table 2:** Effect of a forty-day oral administration of *Corrigiola telephiifolia *root extract in rats.

Dose of CT extract^a^ (mg/kg)	Sex	D/T^b^	Symptoms	Duration of symptoms
0	M	0/8	None	—
	F	0/8	None	—

5	M	0/8	Slight abdominal contractions^c^	—
	F	0/8	Slight abdominal contractions^c^	—

70	M	0/8	(i) Abdominal contractions^c^	—
			(ii) Hypoactivity, hunched posture, loss of appetite, mild diarrhea.	1 week
			(iii) Polyuria, yellow feces.	5 weeks
	F	0/8	(i) Abdominal contractions^c^	—
			(ii) Hypoactivity, hunched posture, loss of appetite, mild diarrhea.	2 weeks
			(iii) Polyuria, yellow feces.	5 weeks

2000	M	0/8	(i) Abdominal contractions^c^	—
			(ii) Hypoactivity, hunched posture, loss of appetite, mild diarrhea.	2-3 weeks
			(iii) Polyuria, yellow feces	5 weeks
	F	0/8	(i) Abdominal contractions^c^	—
			(ii) Marked hypoactivity, hunched posture, loss of appetite, intense diarrhea,	4 weeks
			(iii) Respiratory difficulties for 2 rats	1 week
			(iv) Polyuria, yellow feces	5 weeks

^a^ *C* *orrigiola telephiifolia* extract was administered as a reiterated dose to groups of rats for forty days.

^b^D/T: dead /treated rats.

^c^The abdominal contractions were noted only after the oral administration of the extract.

**Table 3:** Changes in the mean bodyweight of rats after daily oral treatment (40 days) with *Corrigiola telephiifolia* extract.

			*Corrigiola telephiifolia* (mg/kg bodyweight)		
		Control	5	70	2000
Females	Days				
	D1	199.4 ± 4.8	200.3 ± 3.8	216.0 ± 3.5	187.1 ± 3.6
	D5	205.4 ± 4.9	206.4 ± 4.7	214.0 ± 4.2	175.0 ± 3.0^a∗^
	D10	210.0 ± 4.6	210.9 ± 4.5	218.0 ± 5.2	175.1 ± 3.4^a∗^
	D15	210.0 ± 5.9	210.2 ± 5.3	220.0 ± 5.2	176.7 ± 3.0^a∗^
	D20	216.8 ± 6.1^a^	217.1 ± 6.0^a^	227.0 ± 5.6	182.1 ± 3.7*
	D25	220.1 ± 6.6^a^	221.0 ± 6.5^a^	228.0 ± 4.0^a^	185.5 ± 4.1*
	D30	220.3 ± 6.2^a^	221.5 ± 6.3^a^	230.5 ± 5.3^a^	187.7 ± 5.0*
	D35	222.9 ± 4.8^a^	224.1 ± 4.9^a^	231.0 ± 4.8^a^	189.3 ± 4.1*
	D40	221.5 ± 4.9^a^	223.8 ± 5.0^a^	229.0 ± 4.6^a^	190.9 ± 3.3*

Males	Days				
	D1	242.8 ± 2.9	245.1 ± 2.6	247.4 ± 3.5	250.5 ± 2.4
	D5	251.7 ± 3.2	253.6 ± 3.1	246.7 ± 2.8	242.0 ± 2.5^a∗^
	D10	253.2 ± 3.6^a^	256.5 ± 3.2^a^	253.7 ± 3.4^a^	245.6 ± 2.4
	D15	255.5 ± 3.9^a^	259.4 ± 4.0^a^	264.6 ± 4.1^a^	249.1 ± 4.1
	D20	261.2 ± 4.6^a^	264.3 ± 5.1^a^	267.9 ± 5.0^a^	251.6 ± 6.3
	D25	270.7 ± 5.5^a^	274.6 ± 5.3^a^	269.3 ± 5.2^a^	254.3 ± 4.2*
	D30	273.6 ± 6.2^a^	277.1 ± 5.6^a^	274.4 ± 5.2^a^	259.1 ± 3.1^a^
	D35	276.6 ± 7.0^a^	280.3 ± 6.2^a^	277.0 ± 6.1^a^	264.1 ± 3.6^a^
	D40	277.5 ± 6.6^a^	281.2 ± 6.5^a^	277.8 ± 6.0^a^	266.9 ± 3.5^a^

Values are expressed as mean ± SEM. The different superscript letters indicate statistically significant differences (*P* < .05) in the Student's *t*-test.

*as compared with the control group; ^a^as compared with the initial weight of rats on the first day (D1) of the treatment for each group.

**Table 4:** Effect of a forty-day oral administration of *Corrigiola telephiifolia* extract on hematological parameters of Wistar rats.

			*Corrigiola telephiifolia *(mg/kg/day)		
		Control	5	70	2000
Females	Parameter				
	WBC (10^3^/mm^3^)	6.2 ± 0.6	6.0 ± 0.5	5.9 ± 0.5	5.9 ± 0.9
	RBC (10^6^/mm^3^)	6.2 ± 0.4	6.2 ± 0.3	6.5 ± 0.4	6.2 ± 0.3
	HGB (g/dL)	10.7 ± 0.7	10.8 ± 0.6	11.2 ± 0.6	10.3 ± 0.4
	HCT (%)	33.3 ± 2.3	33.5 ± 2.3	35.2 ± 2.3	32.2 ± 1.6
	PLT (10^3^/mm^3^)	143.0 ± 13.5	145.0 ± 15.1	151.7 ± 19.5	146.4 ± 14.2
	MCV (*μ*m^3^)	54.2 ± 1.0	54.2 ± 0.9	54.3 ± 0.6	52.1 ± 0.4
	MCH (pg)	17.4 ± 0.3	17.4 ± 0.2	17.3 ± 0.15	16.6 ± 0.3
	MCHC (g/dL)	32.1 ± 0.2	32.0 ± 0.3	32.0 ± 0.3	32.0 ± 0.3
	RDW (%)	13.2 ± 0.3	13.3 ± 0.3	13.3 ± 0.2	13.7 ± 0.3
	MPV (*μ*m^3^)	9.1 ± 0.8	9.2 ± 0.5	10.2 ± 0.5	9.3 ± 1.0

Males					
	WBC (10^3^/mm^3^)	7.4 ± 1.5	6.8 ± 0.9	6.4 ± 0.7	6.8 ± 0.9
	RBC (10^6^/mm^3^)	6.6 ± 0.3	6.5 ± 0.4	6.4 ± 1.0	7.8 ± 0.9
	HGB (g/dL)	10.8 ± 0.4	10.7 ± 0.8	10.3 ± 1.5	12.2 ± 1.1
	HCT (%)	34.9 ± 1.5	34.5 ± 1.9	32.9 ± 5.4	41.1 ± 4.9
	PLT (10^3^/mm^3^)	158.2 ± 11.3	149.4 ± 13.2	124.8 ± 14.6	136.2 ± 19.2
	MCV (*μ*m^3^)	53.2 ± 0.7	52.3 ± 0.6	51.6 ± 0.8	52.8 ± 0.6
	MCH (pg)	16.5 ± 0.2	16.2 ± 0.2	16.2 ± 0.3	15.8 ± 0.4
	MCHC (g/dL)	31.0 ± 0.4	31.2 ± 0.5	31.6 ± 0.7	30.1 ± 0.8
	RDW (%)	14.0 ± 0.7	13.7 ± 0.4	13.6 ± 0.3	13.8 ± 0.4
	MPV (*μ*m^3^)	7.0 ± 1.5	7.2 ± 0.5	7.5 ± 0.4	8.1 ± 0.4

*C. telephiifolia *extract was administered daily for 40 consecutive days to rats (*n* = 8). Data are expressed as mean ± S.E.M. There were no statistically significant differences between control and treated groups.

**Table 5:** Effect of a forty-day oral administration of *Corrigiola telephiifolia* extract on biochemical parameters of female Wistar rats.

		*Corrigiola telephiifolia* (mg/kg/day)		
	Control	5	70	2000
Parameter				
Na mmol/L	149.4 ± 0.7	148.0 ± 0.5	141.6 ± 1.0*	136.1 ± 1.7*
Cl mmol/L	117.3 ± 0.5	116.4 ± 0.3	110.1 ± 0.7*	106.3 ± 1.1*
K mmol/L	8.5 ± 0.5	8.2 ± 0.6	7.8 ± 0.5	9.3 ± 0.9
Mg mmol/L	1.6 ± 0.2	1.5 ± 0.2	1.5 ± 0.1	1.4 ± 0.1
Ca mmol/L	2.2 ± 0.2	2.1 ± 0.2	2.0 ± 0.1	2.0 ± 0.1
Iron *μ*mol/L	51.6 ± 4.4	52.3 ± 3.7	53.6 ± 2.8	46.7 ± 4.0
Ph.org mmol/L	3.2 ± 0.2	3.3 ± 0.4	3.8 ± 0.5	4.3 ± 0.4*
Total Protein g/L	75.4 ± 1.0	74.1 ± 1.2	70.9 ± 2.0	69.1 ± 1.6*
Glucose mmol/L	8.0 ± 1.0	7.5 ± 0.8	6.7 ± 0.5	6.4 ± 1.0
Urea mmol/L	6.6 ± 0.2	6.3 ± 0.5	5.6 ± 0.5	6.0 ± 0.4
Creatinine *μ*mol/L	26.4 ± 5.1	25.7 ± 1.3	22.9 ± 1.2	87.0 ± 15.6*
Bicarbonates mm Hg	16.0 ± 1.8	15.4 ± 1.1	14.0 ± 1.5	20.9 ± 2.1
Uric acid *μ*mol/L	194.5 ± 59.7	183.7 ± 35.2	177.3 ± 43.6	175.6 ± 50.1
Total cholesterol mmol/L	1.7 ± 0.1	1.7 ± 0.1	1.6 ± 0.1	1.4 ± 0.1
Triglycerides mmol/L	1.8 ± 0.3	1.7 ± 0.2	1.7 ± 0.2	1.3 ± 0.3
Bilirubin *μ*mol/L	25.4 ± 2.5	25.0 ± 2.4	24.4 ± 2.1	27.4 ± 2.9
AST UI/L	54.5 ± 6.1	55.1 ± 6.0	57.7 ± 6.2	78.5 ± 21.8
ALT UI/L	20.2 ± 1.7	21.3 ± 2.5	27.1 ± 5.0	33.7 ± 8.6
ALP UI/L	121.7 ± 10.8	115.9 ± 7.5	103.0 ± 9.9	202.1 ± 30.0*
GGT UI/L	1.4 ± 0.1	1.4 ± 0.2	1.5 ± 0.2	1.9 ± 0.2*

*C. telephiifolia* extract was administered daily for 40 consecutive days to rats (*n* = 8). Data are expressed as mean ± S.E.M.

*Significantly different from the control (*P* < .05).

**Table 6:** Effect of a forty-day oral administration of *Corrigiola telephiifolia* extract on biochemical parameters of male Wistar rats

		*Corrigiola telephiifolia* (mg/kg/day)		
	Control	5	70	2000
Parameter				
Na mmol/L	140.9 ± 0.6	139.5 ± 0.3	138.3 ± 0.6*	135.1 ± 1.3*
Cl mmol/L	111.3 ± 0.6	110.7 ± 0.2	105.0 ± 0.5*	103.4 ± 0.4*
K mmol/L	8.0 ± 0.3	8.0 ± 0.4	8.0 ± 0.5	7.7 ± 0.4
Mg mmol/L	1.5 ± 0.1	1.6 ± 0.1	1.9 ± 0.1	1.4 ± 0.1
Ca mmol/L	1.7 ± 0.1	1.7 ± 0.1	1.9 ± 0.1	1.8 ± 0.1
Iron *μ*mol/L	28.9 ± 1.6	28.7 ± 1.5	28.6 ± 2.2	32.7 ± 2.3
Ph.org mmol/L	4.4 ± 0.4	4.3 ± 0.4	4.1 ± 0.4	4.5 ± 0.5
Total protein g/L	66.4 ± 1.2	66.5 ± 1.0	67.9 ± 1.1	66.6 ± 1.7
Glucose mmol/L	6.6 ± 0.2	6.5 ± 0.3	6.4 ± 0.4	6.9 ± 0.5
Urea mmol/L	5.0 ± 0.3	5.1 ± 0.2	5.1 ± 0.2	4.7 ± 0.3
Creatinine *μ*mol/L	23.2 ± 1.7	23.1 ± 0.9	23.0 ± 0.7	26.8 ± 2.0
Bicarbonates mm Hg	21.3 ± 0.8	21.5 ± 0.7	22.1 ± 1.0	20.6 ± 1.3
Uric acid *μ*mol/L	271.1 ± 29.2	264.8 ± 24.5	251.4 ± 21.4	200.9 ± 35.8
Total cholesterol mmol/L	1.5 ± 0.1	1.5 ± 0.1	1.4 ± 0.1	1.7 ± 0.1
Triglycerides mmol/L	1.2 ± 0.1	1.2 ± 0.1	1.1 ± 0.1	1.2 ± 0.2
Bilirubin *μ*mol/L	27.5 ± 1.3	27.8 ± 1.4	28.1 ± 1.5	31.9 ± 2.0
AST UI/L	77.9 ± 6.9	79.5 ± 7.3	81.9 ± 7.1	114.5 ± 23.5
ALT UI/L	25.0 ± 1.4	24.7 ± 1.5	22.2 ± 1.3	41.3 ± 9.6
ALP UI/L	146.3 ± 23.6	153.6 ± 21.3	164.1 ± 15.8	173.4 ± 28.3
GGT UI/L	1.5 ± 0.2	1.5 ± 0.1	1.4 ± 0.1	2.2 ± 0.4

*C. telephiifolia *extract was administered daily for 40 consecutive days to rats (*n* = 8). Data are expressed as mean ± S.E.M.

*Significantly different from the control (*P* < .05).

**Table 7:** Effect of a forty-day oral administration of *Corrigiola telephiifolia* extract on relative organ weights of Wistar rats.

			*Corrigiola telephiifolia *(mg/kg/day)		
		Control	5	70	2000
Females	Organ				
	Heart	0.31 ± 0.02	0.30 ± 0.03	0.28 ± 0.02	0.31 ± 0.05
	Liver	2.20 ± 0.12	2.25 ± 0.10	2.28 ± 0.10	2.35 ± 0.10
	Spleen	0.26 ± 0.03	0.25 ± 0.01	0.21 ± 0.01	0.22 ± 0.03
	Stomach	0.65 ± 0.10	0.64 ± 0.08	0.63 ± 0.10	0.68 ± 0.09
	Right lung	0.29 ± 0.03	0.3 ± 0.04	0.30 ± 0.03	0.39 ± 0.10
	Left lung	0.24 ± 0.04	0.23 ± 0.03	0.22 ± 0.02	0.27 ± 0.07
	Right kidney	0.31 ± 0.02	0.30 ± 0.02	0.25 ± 0.02	0.29 ± 0.04
	Left kidney	0.29 ± 0.01	0.28 ± 0.01	0.26 ± 0.01	0.28 ± 0.04

Males	Organ				
	Heart	0.32 ± 0.01	0.33 ± 0.01	0.35 ± 0.05	0.30 ± 0.01
	Liver	2.60 ± 0.12	2.70 ± 0.14	2.79 ± 0.16	2.76 ± 0.18
	Spleen	0.23 ±0.01	0.22 ± 0.01	0.21 ± 0.01	0.22 ± 0.01
	Stomach	0.63 ± 0.04	0.64 ± 0.04	0.66 ± 0.04	0.73 ± 0.05
	Right lung	0.30 ± 0.01	0.30 ± 0.02	0.30 ± 0.03	0.31 ± 0.02
	Left lung	0.22 ± 0.01	0.22 ± 0.01	0.20 ± 0.01	0.21 ± 0.02
	Right kidney	0.30 ± 0.01	0.29 ± 0.01	0.28 ± 0.02	0.31 ± 0.01
	Left kidney	0.28 ± 0.01	0.29 ± 0.01	0.27 ± 0.02	0.30 ± 0.01

*C. telephiifolia *extract was administered daily for 40 consecutive days to rats (*n* = 8). Data are expressed as mean ± S.E.M. There were no statistically significant differences in the relative organ weights.
